# Subcritical water extraction of *Equisetum arvense* biomass withdraws cell wall fractions that trigger plant immune responses and disease resistance

**DOI:** 10.1007/s11103-023-01345-5

**Published:** 2023-05-02

**Authors:** Diego Rebaque, Gemma López, Yolanda Sanz, Francisco Vilaplana, Frèderic Brunner, Hugo Mélida, Antonio Molina

**Affiliations:** 1 Centro de Biotecnología y Genómica de Plantas, Universidad Politécnica de Madrid (UPM) - Instituto Nacional de Investigación y Tecnología Agraria y Alimentaria (INIA/CSIC), Pozuelo de Alarcón (Madrid), Campus de Montegancedo UPM, Madrid, 28223 Spain; 2 Departamento de Biotecnología-Biología Vegetal, Escuela Técnica Superior de Ingeniería Agronómica, Alimentaría y de Biosistemas, UPM, Madrid, 28040 Spain; 3 PlantResponse Inc, Centro de Empresas, Campus de Montegancedo UPM, 28223-Pozuelo de Alarcón (Madrid), Madrid, Spain; 4 Division of Glycoscience, School of Engineering Sciences in Chemistry, Biotechnology and Health, Royal Institute of Technology (KTH), Stockholm, Sweden; 5 Área de Fisiología Vegetal, Departamento de Ingeniería y Ciencias Agrarias, Universidad de León, León, Spain

**Keywords:** Cell wall, Disease resistance, Glycan, Pattern triggered immunity (PTI), Subcritical Water extraction (SWE)

## Abstract

Plant cell walls are complex structures mainly made up of carbohydrate and phenolic polymers. In addition to their structural roles, cell walls function as external barriers against pathogens and are also reservoirs of glycan structures that can be perceived by plant receptors, activating Pattern-Triggered Immunity (PTI). Since these PTI-active glycans are usually released upon plant cell wall degradation, they are classified as Damage Associated Molecular Patterns (DAMPs). Identification of DAMPs imply their extraction from plant cell walls by using multistep methodologies and hazardous chemicals. Subcritical water extraction (SWE) has been shown to be an environmentally sustainable alternative and a simplified methodology for the generation of glycan-enriched fractions from different cell wall sources, since it only involves the use of water. Starting from *Equisetum arvense* cell walls, we have explored two different SWE sequential extractions (isothermal at 160 ºC and using a ramp of temperature from 100 to 160 ºC) to obtain glycans-enriched fractions, and we have compared them with those generated with a standard chemical-based wall extraction. We obtained SWE fractions enriched in pectins that triggered PTI hallmarks in *Arabidopsis thaliana* such as calcium influxes, reactive oxygen species production, phosphorylation of mitogen activated protein kinases and overexpression of immune-related genes. Notably, application of selected SWE fractions to pepper plants enhanced their disease resistance against the fungal pathogen *Sclerotinia sclerotiorum*. These data support the potential of SWE technology in extracting PTI-active fractions from plant cell wall biomass containing DAMPs and the use of SWE fractions in sustainable crop production.

**Supplementary Information:**

The online version contains supplementary material available at 10.1007/s11103-023-01345-5.

## Key message

Subcritical water extraction of *Equisetum arvense* cell wall generates glycan-enriched fractions that contain Damage Associated Molecular Patterns (DAMPs) that trigger plant immune responses and disease resistance.

**Supplementary Information:**

The online version contains supplementary material available at 10.1007/s11103-023-01345-5.

## Introduction

Over the course of evolution, plants have developed strong resistance mechanisms against pathogen infections. However, since specialized pathogens have coevolved different sets of virulence mechanisms to overcome plant disease resistance and to colonise plants, they still cause important crop yield losses yearly (Savary et al. 2017; Strange and Scott 2005). In addition to structural defensive mechanisms, plants have developed a cell-autonomous and complex multi-layered immune system (Gust et al. 2017; Roland and Beutler 2010; Zipfel 2014). One of these immunity layers perceives “non-self” molecular patterns derived from pathogens (Microbe Associated Molecular Patterns, MAMPs) or “self” patterns derived from plants (Damage Associated Molecular Patterns, DAMPs) upon plant damage produced during its colonization by pathogens or pests attack (Boller and Felix 2009; Jones and Dangl 2006). Plants are able to recognize DAMPs and MAMPs through plasma membrane anchored Pattern Recognition Receptors (PRRs), which activate a first layer of defence, known as Pattern Triggered Immunity (PTI). PTI involves several molecular events like cytoplasmic calcium influxes, production of reactive oxygen species (ROS), phosphorylation of mitogen activated protein kinases (MPKs) and up-regulation of the expression of immune-related genes (Bigeard et al. 2015). It has been demonstrated that pre-treatment of *Arabidopsis thaliana* plants and crops with MAMPs/DAMPs prior pathogen inoculation or colonization confers enhanced disease resistance in comparison to untreated plants (Boutrot and Zipfel 2017; Claverie et al. 2018; Lorenzo and Cervone 2022; Mélida et al., 2020, Rebaque et al. 2021; Zarattini et al. 2021). Accordingly, MAMPs/DAMPs could be used as novel biological products for crop protection or physiological biostimulation, which will activate crop immune responses and disease resistance mechanisms, contributing to a more sustainable agriculture by replacing chemical pesticides. Crops treated with these immune-active MAMPs/DAMPs will have an enhanced capacity to perceive pathogens and to activate defensive responses faster than untreated crops (Conrath et al. 2015; Mauch-Mani et al. 2017).

Peptidic bacterial MAMPs are the best-characterized ligands triggering PTI. Particularly, flagellin- (flg22) and elongation factor Tu-(elf18)-derived peptides have been long studied, and the mechanisms for their perception are relatively well understood compared with others MAMPs (Boutrot and Zipfel 2017). However, the possibility of developing crop protection technologies based on the use of these or other peptidic MAMPs is far from being feasible due to the very high cost of their large-scale production. In this context, it is interesting to bear in mind that plant and microbial cell walls are source of complex structures that could be perceived as MAMPs/DAMPs by plant cells (Ayers et al. 1976; Aziz et al. 2007; Bacete et al. 2018, 2020; Cabrera et al. 2006; Claverie et al. 2018; Côté and Hahn 1994; Desaki et al. 2018; Mélida et al. 2018, 2020; Miya et al. 2007; Nars et al. 2013; Rebaque et al. 2021; Yang et al. 2021; Voxeur et al. 2019; Willmann et al. 2011; Zang et al. 2019; Martín-Dacal et al. 2023). Plant dry material (biomass) consist mainly of cell walls whose composition vary between species and tissues, but are mainly made up of phenolic (lignin) and carbohydrate polymers (e.g. cellulose, hemicelluloses and pectins). The complexity, abundance, and structural and compositional diversity of cell walls have led to consider them as resource for different industrial processes and products (Kumar et al. 2021; Ekielski and Mishra 2020; Rebaque et al. 2017). After the initial discoveries in the 1980s of cell wall derived-DAMPs in plants (initially known as oligosaccharins; Fry 1994), the recent finding of several new glycans perceived as DAMPs has increased the interest of using glycans in crop protection. In this direction, the plant cell walls are endless sources of immune active glycans, like pectin-derived oligogalacturonides (OGs) (Voxeur et al. 2019), cellulose-derived β-1-4 glucans (Aziz et al. 2007; Souza et al. 2017), arabinoxylans (Mélida et al. 2020) or β-1-3/β-1-4 (mixed- linked) glucans (MLGs; Rebaque et al. 2021; Yang et al. 2021; Barghahn et al. 2021; Martín-Dacal et al. 2023).

The use of DAMPs extracted from low-value plant biomass in agriculture could be interesting for the development of products aligned with the concept of the “green circular economy”. Some of these DAMPs have been shown to be extractable from plant cell walls by using established chemical methods which are based on the interaction between chemical solutions and the various bonds linking the wall polymers to each other (Bacete et al. 2017, 2020; Molina et al. 2021). Those standardized and chemical-based extraction methods require the use of chelating agents (e.g. 1,2-cyclohexylenedinitrilo-tetraacetic acid), basic (e.g. KOH, NaOH, Na_2_CO_3_) or acid solutions (e.g. HCl, trifluoroacetic acid) and reducing agents (e.g. NaBH_4_) (Bacete et al. 2017). However, these chemical products are labelled as corrosive, health hazardous, irritating and toxic, and biological extracts containing them cannot be released to the environment due to their harmful impact. Moreover, the use of these hazardous chemicals for the extraction of cell wall constituents generally implies long additional steps of dialysis for their removal if a further biochemical characterization is planned or if these cell wall fractions are the biological components used to generate agronomic products. These additional steps to remove the chemicals from wall fractions increase the hazardous wastes and costs of the final products, narrowing the usability of these wall-derived fractions. Aqueous extractions of poly- and oligosaccharides from cell wall enriched biomass would be an alternative extraction methodology, since it will avoid the use of these chemical compounds. In fact, as a proof of concept, aqueous extractions at room temperature obtained from cell walls of *A. thaliana ccr1* mutant impaired in lignin biosynthesis, leaded to the identification of DAMPs probably containing galacturonic acid (Gallego-Giraldo et al. 2018, 2020). Interestingly, subcritical water extraction (SWE) has been described as an environmentally-friendly method to extract polymers from cell walls by just using water at high temperatures and under high-pressure conditions, based on the alteration of the physio-chemical properties of water, such as its dielectric constant, viscosity and diffusion, produced at subcritical conditions, in contrast to extraction based on the breaking of phenolic compounds linkages produce by alkaline traditional treatments (Rudjito et al. 2019; Rincón et al. 2021). SWE has been proved to be effective in the extractions of pectins from fruits peel (Ueno et al. 2008; Wang et al. 2014), β-glucans from *Ganoderma lucidum*, barley or oyster mushroom (Jo et al. 2013; Kodama et al. 2016), and feruloylated arabinoxylans (Rudjito et al. 2019). However, the use of SWE to obtain fractions enriched in MAMPs/DAMPs from cell wall material and the capacity of such fractions to protect plants from pathogen infections have not been tested in detail.

In a previous work, we used a hemicellulosic fraction (KOH) from *Equisetum arvense* (horsetail) cell walls as a possible source material to obtain MLG oligosaccharides, although the yield was lower than using fungal material as a source (Fry et al. 2008; Rebaque et al. 2021). However, this cell wall material turned out to be a convenient source of glycan-enriched biomass with interesting features. Here, we have explored the potential of SWE to obtain DAMPs from *E. arvense* cell walls and compared it with a standard alkali extraction methodology. Indeed, we show here that *E. arvense* SWE fractions obtained were active in modulating PTI responses and triggering disease resistance in plants. Thus, our data support the benefits of SWE to extract active glycans from different sources, which could be easily scaled-up to develop novel products from cell wall-enriched biomass to enhance crop disease resistance.

## Materials and methods

### Biological materials and growth conditions

*A. thaliana* Columbia-0 (Col-0) background lines were used for all the experiments in the present work. *A. thaliana* seedlings used for Ca^2 +^ _cyt_ (Col-0^AEQ^; Knight et al. 1991; Ranf et al. 2011), MAPKs phosphorylation and gene expression analyses were grown in liquid MS medium and *A. thaliana* plants for ROS analyses in soil-vermiculite (3:1) under 10 h light/14 hours dark conditions at 21 − 20 ºC (Mélida et al. 2018). Pepper plants (*Capsicum annuum*, Murano) were grown in greenhouse in soil-vermiculite (3:1) under 14 h of light/10 hours of dark at 24 − 19 ºC. *Sclerotinia sclerotiorm* isolate was refreshed from Plant Response Inc. mycelia stock collection on solid potato dextrose agar (PDA) at 24 ºC for 7 days in the dark. For preparing the inoculum, two agar discs (1 cm^2^) were transferred to 150 ml of potato dextrose broth PDB and grown at 24 ºC under shaking (75 rpm) for 7 days before inoculation (Chen and Wang 2005).

### MAMP/DAMP used in the experiments

Chitohexaose (β-1,4-D-(GlcNAc)_6_; #O-CHI6) and MG43 (β-1,4-D-(Glc)_2_-β-1,3-D-Glc, # O-BGTRIB) were purchased from Megazyme and flg22 was provided by EZBiolab.

### Preparation and fractionation of cell walls of *E. arvense*

**Fig. 1:**
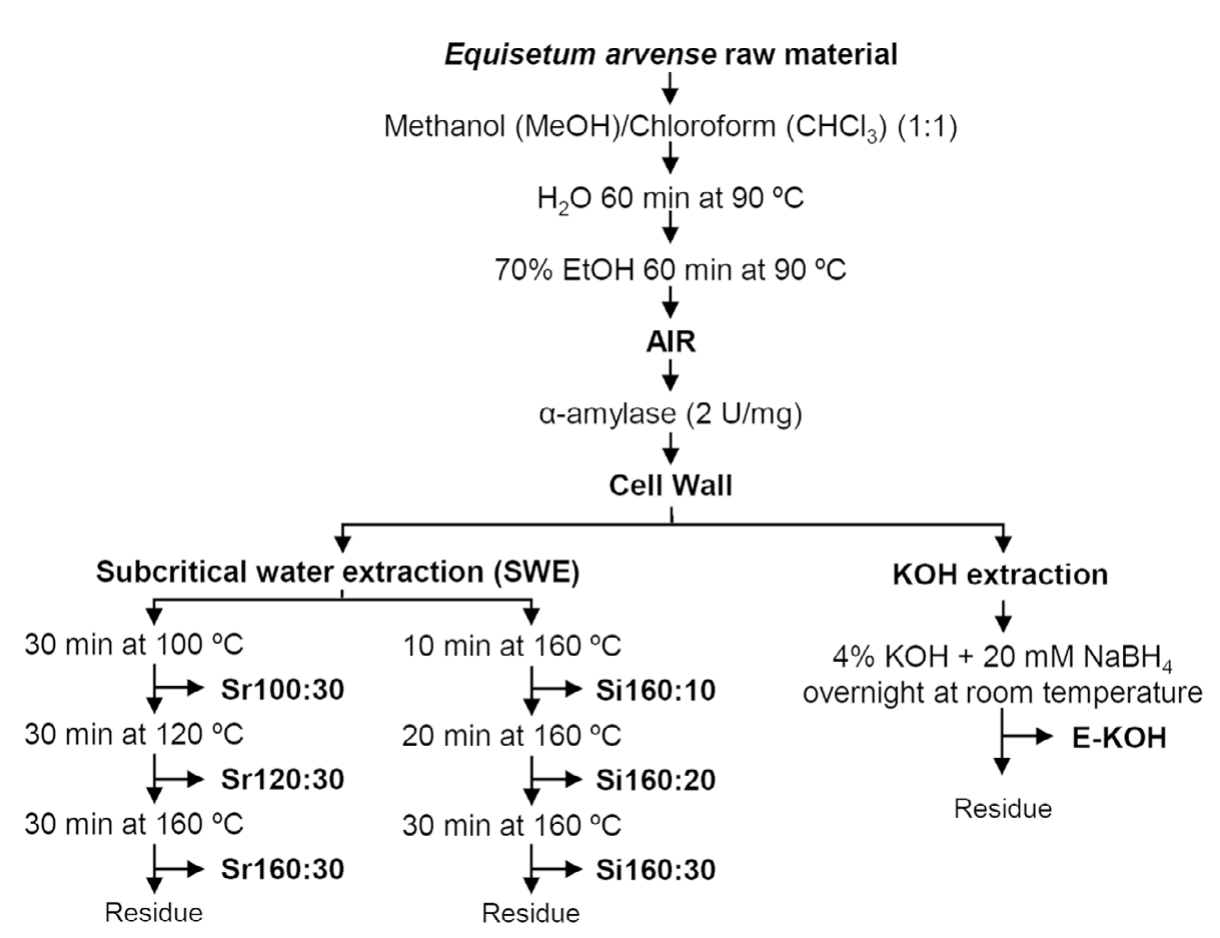
Scheme of the isolation of *Equisetum arvense* cell wall fractions using different subcritical water extraction (SWE) or alkali (KOH) extraction methodologies

*E. arvense* raw material (#COPMCOLO001) from Biosearch Life (Granada, Spain) was fine-powdered using a kitchen blender and extracted with MeOH/CHCl_3_ (1:1) four times during 4 h at 4 ºC (Rebaque et al. 2021). Vacuum pump filtration was used to separate the soluble fraction after each step. Soluble fractions were discarded, and the insoluble residues were extracted with 70% (v/v) ethanol twice (1 h at 90 ºC and overnight at room temperature). The residue after filtration was then treated with distilled water twice (1 h at 90 ºC and overnight at room temperature). The residue after filtration was considered the Alcohol Insoluble Residue (AIR) (Fig. 1). In order to remove the starch from AIR, 100 mg/ml of material were enzymatically-digested with 2.5 U/ml α-amylase obtained from porcine pancreas (Sigma type VI-A) in 0.01 M phosphate buffer (pH 7.0) for 24 h at 37 ºC. Residue was washed with 70% ethanol and acetone and the filtered and dried final residue was considered as cell wall powder (Rebaque et al. 2017) and was used as starting material for different fractionation methods in triplicate.

Isolated cell wall powder was incubated overnight at room temperature in 4% KOH solution with 20 mM NaBH_4_ (100 ml/g) and centrifuged at 4,400 g for 10 min at room temperature. Supernatant pH was adjusted to 6.0 using glacial acetic acid and dialyzed against deionized water to remove solutes of a small molecular mass (Spectra/Por MWCO 1000 Daltons, Spectrum Laboratories). The final freeze-dried material was the E-KOH fraction.

SWE was performed using an accelerated solvent extractor Dionex ASE 350 (Thermo Fisher Scientific). Three grams of isolated cell wall powder were mixed with 3 g of Dionex ASE Prep DE Diatomaceous Earth (Thermo Fisher Scientific) and placed between ASE.

Extraction Cellulose Filters (Thermo Fisher Scientific) into a 34 ml extraction capsule (Rudjito et al. 2019). Two parallel cell wall extraction sequences were performed (Fig. 1). One sequence was performed under static mode at isothermal conditions (Si) using distilled water at 160 °C for 10, 30, or 60 min. The extractions recovered were named as Si160:10, Si160:20 and Si160:30 respectively (Fig. 1). A parallel sequential extraction was carried out under static mode using a ramp of temperature (Sr). Distilled water was used at 100 ºC for 30 min, 120 ºC for 30 min or 160 ºC for 30 min (Fig. 1). The extractions were recovered and named as Sr100:30, Sr120:30 and Sr160:30 respectively. Steel cells containing the samples were introduced in the oven once the set temperature was reached. In the ramp of temperature process, the cells were placed outside the oven while the next set temperature was reached. The time required for the oven to reach each temperature stage is variable and always lower than 5 min. Fractions were freeze-dried and stored at room temperature until they were used.

For proteinase K (EC 3.4.21.64) digestions, samples (1 mg/ml) were treated with 1 U/ml of Proteinase-K (Omega Bio-tek) overnight at 37 ºC. Afterwards, reactions were stopped by heating for 20 min at 100 ºC.

### SWE and KOH fractions characterization

Total sugar amount was determined by the colorimetric phenol-sulphuric acid method described by Dubois et al. (1956). For monosaccharide analysis, dried purified cell wall fractions (0.5 mg) were hydrolysed in the presence of 2 M trifluoroacetic acid (TFA) at 121 ºC for 3 h. Myo-inositol was used as an internal standard. The resulting monosaccharides were analysed by HPAEC-PAD (Dionex ICS 6000 system; Thermo Fisher Scientific) on a CarboPac PA1 anion exchange column (2 × 250 mm; Thermo Fisher Scientific). Monosaccharides were eluted at 1 ml/min using a linear saline gradient of 100 mM NaOH to 100 mM NaOH/300 mM sodium acetate over 20 min (Ruthes et al. 2017). Three technical replicates were analysed in each of the three fractionation replicates performed.

Total protein quantification in the SWE and KOH fractions was performed by Bradford assay (Bio-Rad). Two technical replicates of two independent fractionation replicates were analysed.

### Calcium influxes measurement

*A. thaliana* 8-days-old MS liquid-grown seedlings of Col-0^AEQ^ were used for cytoplasmic calcium (Ca^2 +^ _cyt_) measurements using the method previously described (Bacete et al. 2017; Mélida et al. 2018) and a Varioskan Lux Reader (Thermo Fisher Scientific). Four to eight seedlings (biological replicates) were analysed in each experiment.

### Reactive oxygen species production

Four mm diameter leaf-discs from five-week-old *A. thaliana* plants grown in sterile soil-vermiculite (3:1) were used to determine H_2_O_2_ production after treatment with wall fractions/MAMPs/DAMPs as previously described (Mélida et al. 2018). ROS production was measured by determining the luminescence produced by luminol (L-012 FUJIFILM Wako Chemicals)-peroxidase reaction (Sang and Macho 2017) in a Varioskan LUX Reader (Thermo Scientific). Eight biological replicates were analysed in each experiment.

### Immunoblot analysis of MAPK activation

Twelve-day-old MS liquid-grown seedlings were treated with water (mock), different cell wall fractions or chitohexaose for 0, 5, 10 and 20 min, and then harvested in liquid nitrogen. Seedlings were homogenized using FastPrep Bead Beating Systems (MP Biomedicals) in extraction buffer (50 mM Tris-HCl pH 7.5, 200 mM NaCl, 1 mM EDTA, 10 mM NaF, 2 mM sodium orthovanadate, 1 mM sodium molybdate, 10% (v/v) glycerol, 0.1% (v/v) Tween-20, 1 mM 1,4-dithiothreitol, 1 mM phenylmethylsulfonyl fluoride, and phosphatase inhibitor cocktail P9599 (Sigma)). Total protein extracts were quantified by Bradford assay (Bio-Rad). Equal amounts of proteins were separated on Mini-PROTEAN TGX, 10%, 10-well, 30 µl (Bio-Rad) gel and transfer to nitrocellulose membrane using Invitrogen™ iBlot™ 2 Gel Transfer Device. Membranes were blocked with Protein-Free (TBS) Blocking Buffer (Thermo Scientific; Pierce) for 2 h at room temperature. Membranes were incubated overnight at 4 ºC in TBS containing Phospho-p44/42 MAPK (Erk1/2) (Thr202/Tyr204) antibody (Cell Signaling Technology) (Chung and Sheen 2017). Membranes were cleaned four times with TBS containing 0.1% Tween-20 and incubated with horseradish peroxidase-conjugated anti-rabbit antibody (GE-Healthcare) (1:5000) in TBS. Membranes were cleaned again and revealed by ECL Western Blotting Substrate (Thermo Scientific; Pierce) and detected using iBright FL1000 Image System (ThermoFisher Scientific). As load control membranes were stained with Ponceau-S Red (Sigma Aldrich).

### Gene expression analyses

Twelve-day-old seedlings grown on liquid MS medium were treated with either chitohexaose, water (mock) or *E. arvense* fractions for 0 and 30 min. Total RNA was extracted with the RNeasy Plant Mini Kit (Qiagen) according to the manufacturer’s protocol. qRT-PCR analyses were performed as previously reported (Bacete et al. 2020). *UBC21* (*At5g25760*) expression was used to normalize the transcript level in each reaction. Oligonucleotides used for qRT-PCR are indicated in Supplementary Table 1. Three technical replicates of ten pulled seedlings were analysed.

### Crop protection assay

Pepper plants (*C. annuum*, Murano) were grown in greenhouse in soil-vermiculite (3:1) under 14 h of light/10 hours of dark at 24 − 22 ºC. Leaves of five-weeks-old plants were spray-treated with 2 ml per plant of cell wall extracts (0.125 mg/ml) or the active glycan MLG43 (Rebaque et al. 2021). Two-days after treatment, plants were moved to a 75% humidity greenhouse chamber and spray-inoculated with 5 ml of a 250 cfu/ml suspension of *S. sclerotiorum* homogenized mycelia according to Chen and Wang (2005). Disease symptoms were determined at 5 and 9 days post-inoculation (dpi) in all the leaves of each plant (n = 24) using a scale from 0 to 5: 0 = no symptoms; 1 = small necrotic spots (< 10% of leaf area); 2 = two or more notable necrotic spots (10–25% of leaf area); 3 = big necrotic area (25–50% of leaf area); 4 = more than 50% of leaf area affected, 5 = leaf fully necrotized (Rebaque et al. 2021).

## Results

### Subcritical water extractions of plant cell walls yield glycan-enriched fractions with different carbohydrate composition

**Fig. 2:**
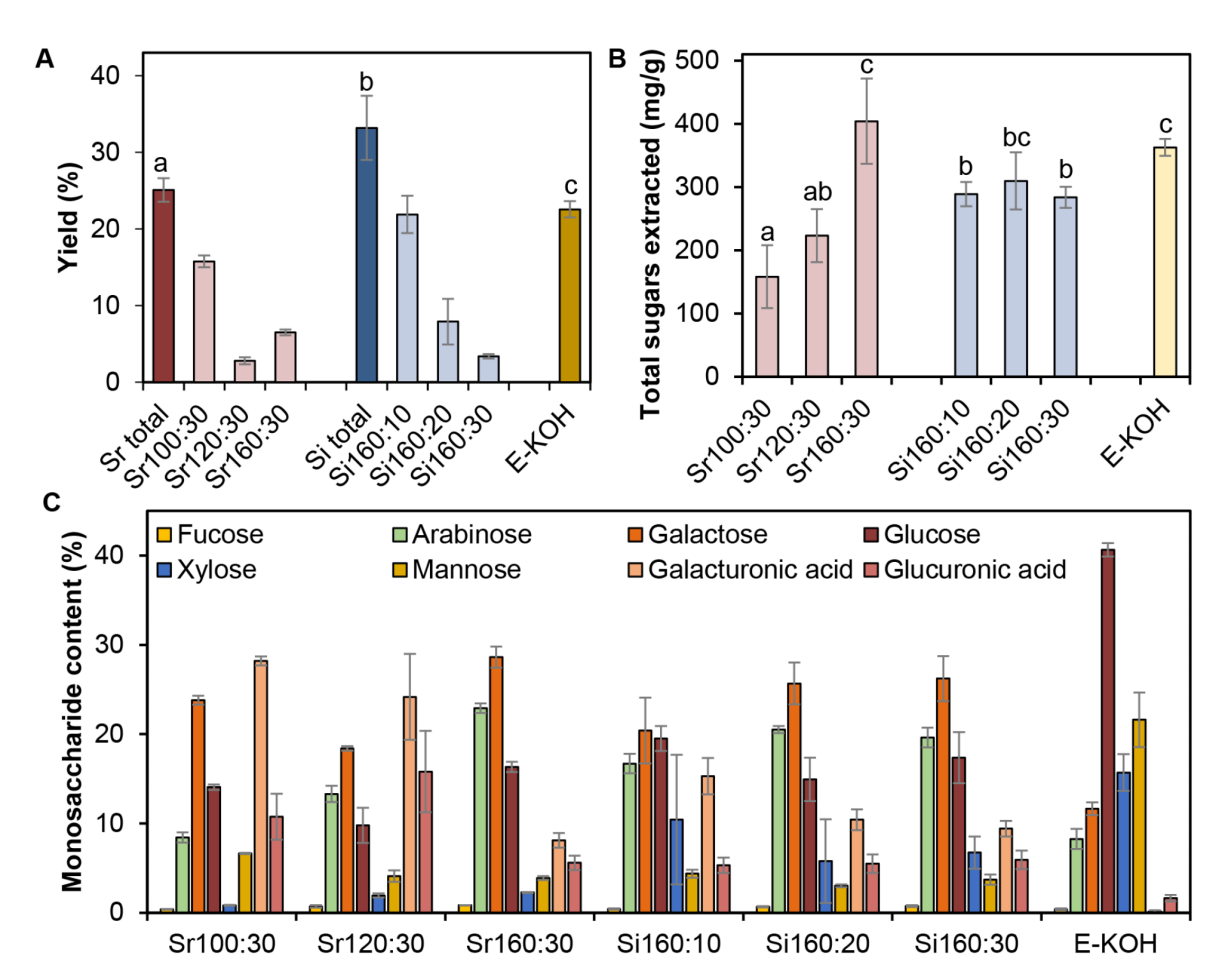
*Equisetum arvense* cell wall fractions analysis. Fractions were obtained by different extraction methods. Fractions obtained after a ramp of temperatures [30 min at 100 ºC (Sr100:30), 30 min at 120 ºC (Sr120:30) and 30 min at 160 ºC (Sr160:30)], or SWE isothermal fractionation [160 ºC 10 min (Si160:10), 20 min (Si160:20) and 30 min (Si160:30)]. Chemical fractions were obtained by incubation with 4% KOH (E-KOH). **A** Total extraction yield as dry weight of extraction relative to initial amount **B** total sugar proportion and **C** monosaccharide analyses expressed as percentage of total sugar are represented. Data represent mean ± SD (n = 3 independent extractions). Different letters indicate significant differences by Student’s t-test (*p* < 0.05)

We used *E. arvense* cell walls as raw material to test the effectiveness of SWE in extracting PTI-active glycans and compared this methodology with a standard KOH cell wall fractionation procedure (Bacete et al. 2017; Rebaque et al. 2017). Using an established methodology (Fry et al. 2008), cell walls were purified from *E. arvense* raw material and then subjected either to chemical 4% KOH extraction (E-KOH) or to SWE using different conditions: isothermal (Si) extraction at 160 ºC for different times (10, 20 and 30 min) or extraction with a sequential temperature ramp (Sr: 100 ºC, 120 ºC and 160 ºC) for 30 min each (Fig. 1). The efficiency in glycans extraction of the different SWE conditions tested was determined by freeze-drying the material extracted and determining the mg obtained per gram of cell wall raw material. Total dry material extracted with SWE ranged between 25 and 33% (w/w) of initial cell wall material for Sr and Si, respectively, yields that were higher than that obtained with KOH extraction (~ 22% w/w; Fig. 2A). The Si extraction at 160 ºC produced higher total yields than Sr extraction using a ramp of temperatures from 100 ºC to 160 ºC (Fig. 2A). Interestingly, the first fractions obtained either using Sr (Sr 100:30) or Si (Si 160:10) yielded more material (~ 16% and 22%, respectively) than the subsequent fractions obtained at higher temperatures (120 ºC and 160 ºC for Sr) or longer extraction periods (30 and 60 min for Si). The amount (% w/w) of extracted dry material with Sr 100:30 or Si 160:10 treatments were similar to those obtained with E-KOH extraction (Fig. 2A). The proportion of carbohydrates in the fractions was determined and found to be higher in SWE fractions obtained at the highest temperature and longest time of extraction (Sr160:30, Fig. 2B), that contained approximately 40% of carbohydrates of its dry weight, a proportion that was higher than those obtained at 100 ºC (Sr100:30) and 120 ºC (Sr120:30) with 16% and 22%, respectively (Fig. 2B). The Si fractions had a sugar content close to 30% in all cases (Fig. 2B). Extraction with alkali produced a good yield in terms of sugar proportion (36%; Fig. 2B), however, requirement of dialysis step after KOH chemical extraction is a major drawback since a large part of the oligosaccharides, the DAMPs ligands of interest, are probably lost during dialysis, and poses an added technical difficulty when it comes to scaling up the process.

Monosaccharide analysis of SWE fractions pointed to an important presence of arabinans, galactans, galacturonans and glucans as indicated by a high proportion of arabinose, galactose, galacturonic acid and glucose (Fig. 2C). In Sr fractions, the arabinose (ranging from 8.4 to 22.9%) and galactose content (ranging from 18.4 to 28.6%) pointed to an increase on arabinogalactan proportion in parallel to the increase in temperature, while galacturonic acid decreased from 28.2 to 8.1%. Interestingly, glucuronic acid was also extracted in all SWE fractions, ranging from 5.3 to 15.8% (Fig. 2C). The extraction of glucose-containing carbohydrates resulted more effective at 160 ºC than in those fractions obtained at lower temperatures, showing values between 9.8% and 19.5% (Fig. 2C). On the other hand, according to our results, 4% KOH extraction yielded the highest proportion of glucose in the extracted fraction, which accounted for 40.7% of the total monosaccharide content of this fraction (Fig. 2C). Alkali also extracted arabinans and galactans (19.9%), but to a lower proportion than SWE, and both uronic acids were detected only at very minor proportions (Fig. 2C). In contrast, xylose and mannose were detected in much higher proportions compared to the SWE and accounted for 15.7% and 21.6% respectively (Fig. 2C). These data indicate that SWE (Si and Sr) of plant cell walls yield glycan-enriched fractions with different carbohydrate composition than that obtained with KOH extraction.

### *E. arvense* cell wall fractions obtained by SWE trigger calcium influxes in *A. thaliana*

**Fig. 3:**
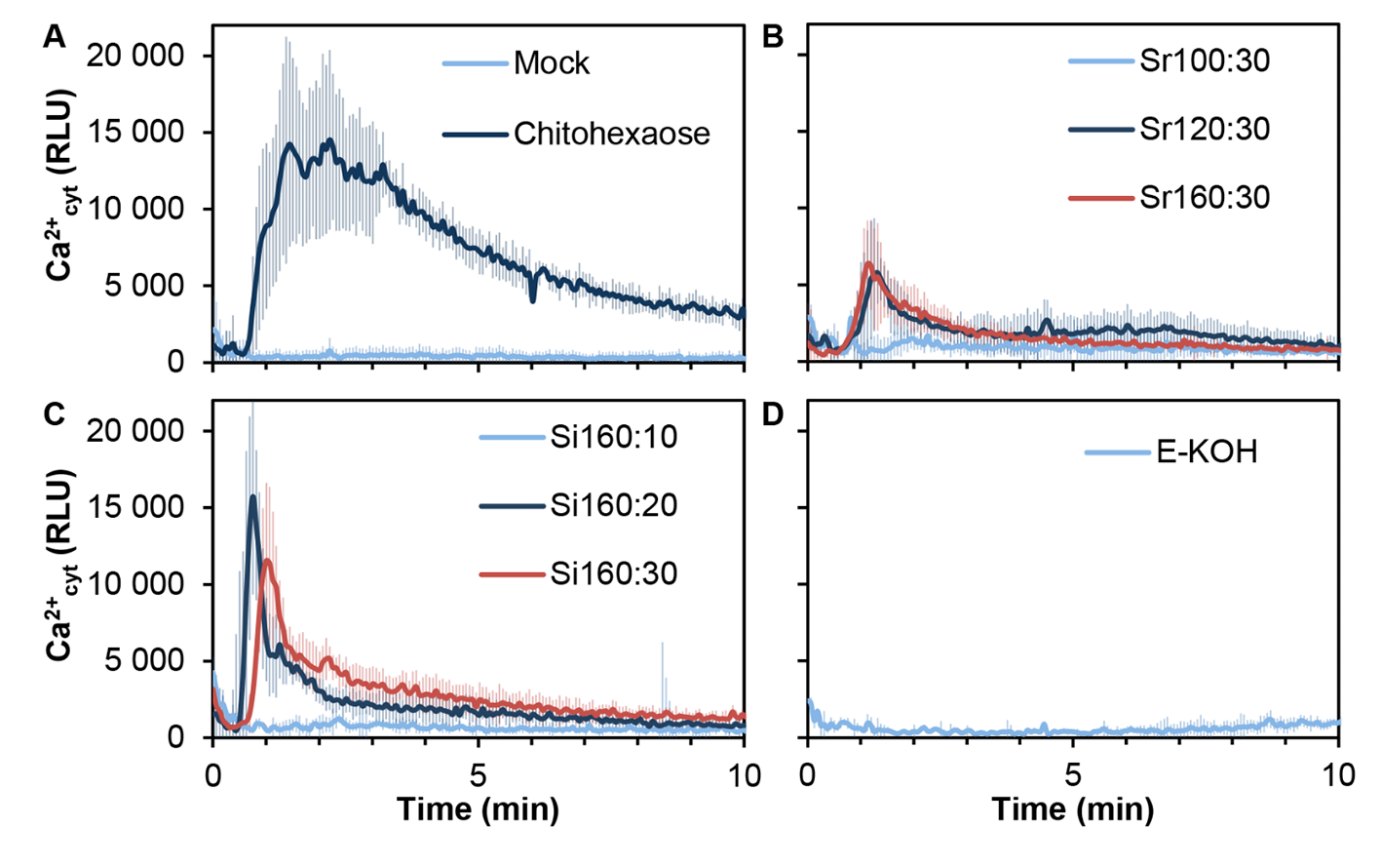
Subcritical water extractions of *Equisetum arvense* cell walls trigger cytoplasmic calcium elevations in*Arabidopsis thaliana*. Calcium influxes measured as relative luminescence units (RLU) over the time in 8-days old *A. thaliana* Col-0^AEQ^ seedlings after treatment with **A** distilled water (mock) and 50 µM chitohexaose, **B** 0.25 mg/ml of SWE fractions obtained after a ramp of temperatures [30 min at 100 ºC (Sr100:30), 30 min at 120 ºC (Sr120:30) and 30 min at 160 ºC (Sr160:30)], **C** 0.25 mg/ml of SWE isothermal fractionation [160 ºC 10 min (Si160:10), 20 min (Si160:20) and 30 min (Si160:30)] and **D** 0.25 mg/ml of a chemical fraction obtained by incubation with 4% KOH (E-KOH). Data represent mean ± SD (n = 8 biological replicates) in all panels. Data shown are from one representative experiment of the three carried out

Determination of calcium influxes upon treatment of *A. thaliana* Col-0^AEQ^ with SWE and E-KOH fractions was used as a first readout method to monitor early PTI responses triggered by the fractions (Knight et al. 1991; Ranf et al. 2011; Mélida et al. 2018). The previously characterised MAMP chitohexaose was used as positive control and water (mock) was included as negative control in these studies (Mélida et al. 2018; Fig. 3A). Most of SWE fractions induced calcium influxes in Col-0^AEQ^ (Fig. 3B, C) that were of a lower intensity than that observed in chitohexaose-treated plants (Fig. 3A). Fractions corresponding to extractions at higher temperatures (Si160:20, Si160:30, Sr120:30 and Sr160:30) produced luminescence peaks more intense than the rest of fractions tested, indicating that they triggered stronger calcium bursts (Fig. 3B, C). This intensity of calcium influxes contrasted with the lack or null activity of SWE fractions obtained at low temperature (Sr100:30) or during short time of extractions at high temperature (Si160:10; Fig. 3B, C). E-KOH fraction was not able to induce calcium influxes in accordance with previous data (Fig. 3Rebaque et al. 2021).

**Fig. 4:**
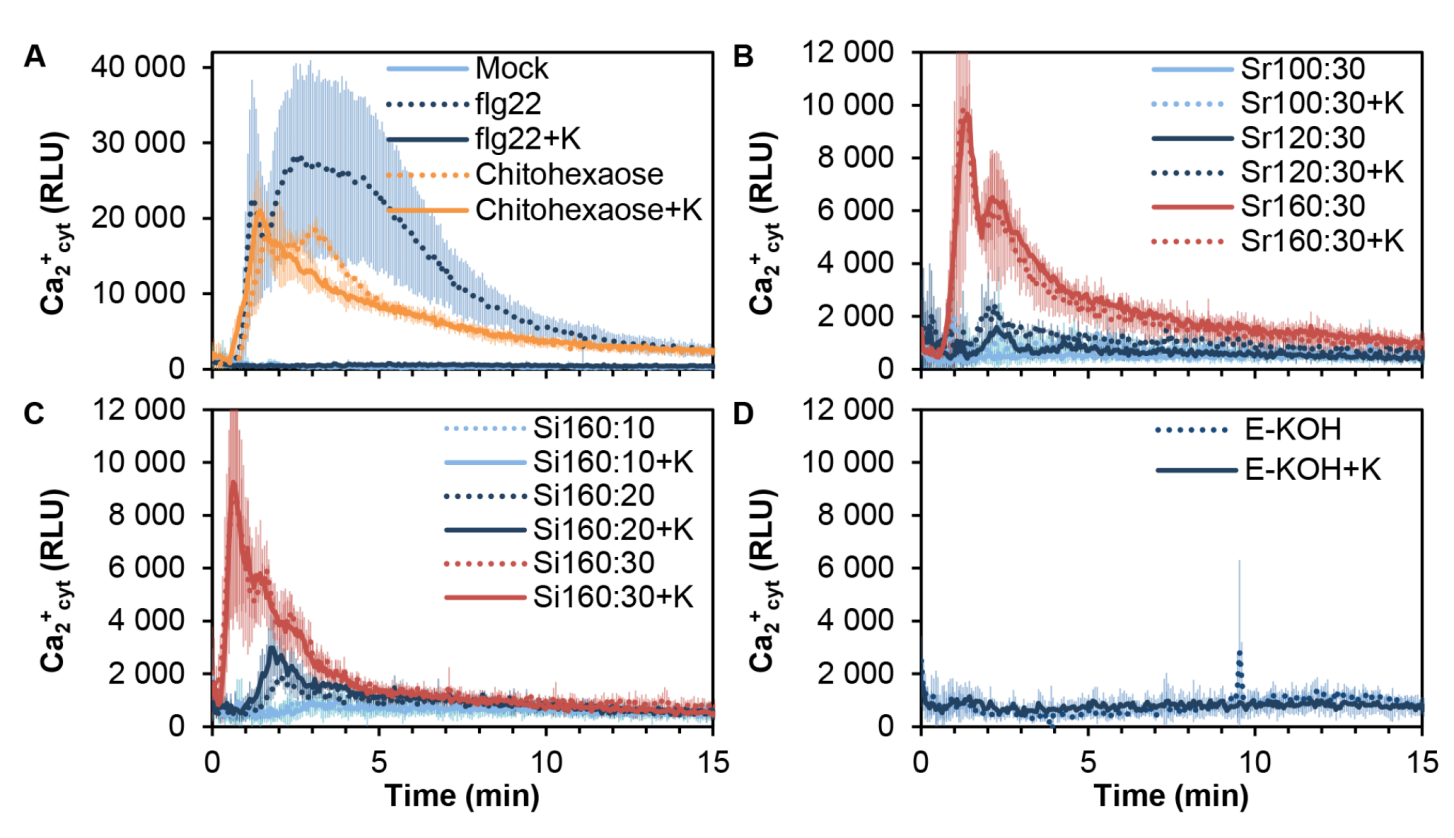
Calcium influxes triggered in *Arabidopsis thaliana* by *Equisetum arvense* SWE fractions are not impaired by their treatment with proteinase-K. Calcium influx measured as relative luminescence units (RLU) over the time in 8-days old *A. thaliana* Col-0^AEQ^ seedlings after treatment with proteinase K (+ K): **A** distilled water (mock), 50 nM flg22 and 50 µM chitohexaose, **B** 0.25 mg/ml of SWE fractions obtained after a ramp of temperatures during 30 min at 100 ºC (Sr100:30), 120 ºC (Sr120:30) and 160 ºC (Sr160:30), **C** 0.25 mg/ml of SWE fractions obtained after at isothermal 160 ºC during 10 min (Si160:10), 20 min (Si160:20) and 30 min (Si160:30) fractions and **D** 0.25 mg/ml of 4% KOH fractionation (E-KOH). Samples in panels b-d were preheated 10 min at 100 ºC. Data represent mean (n = 8 biological replicates). Data shown are from one representative experiment of the two performed

To exclude that the calcium bursts triggered by these fractions would be mediated by proteins or peptides present in SWE and KOH fractions, they were treated with proteinase-K and their activity on Col-0^AEQ^ tested (Fig. 4). In these experiments, in addition to chitohexaose the MAMP peptide flg22 was used as control, since its PTI activity is lost after proteinase treatment (Fig. 4; Mélida et al. 2020). We found that calcium influxes observed were similar either for proteinase-K-treated or -untreated SWE fractions, revealing that SWE fractions activity was not inactivated by proteinase treatment (Fig. 4B,C), further demonstrating that the active DAMPs of these fractions are not of proteinaceous nature. As expected, E-KOH fractions treated with proteinase remained unable to trigger calcium influxes (Fig. 4D) whereas flg22 treated with proteinase-K lost its PTI activity (Fig. 4A). We also determined the total proteins present in these fractions and found that they contained a very low proportion of proteins (Supplementary Fig. 1) in comparison to the carbohydrate’s counterpart (Fig. 2A). These data suggest that the active ligands of the cell wall fractions triggering calcium bursts in *A. thaliana* Col-0^AEQ^ were not proteins/peptides.

### *E. arvense* cell wall Si/Sr fractions trigger PTI hallmarks in *A. thaliana*

**Fig. 5:**
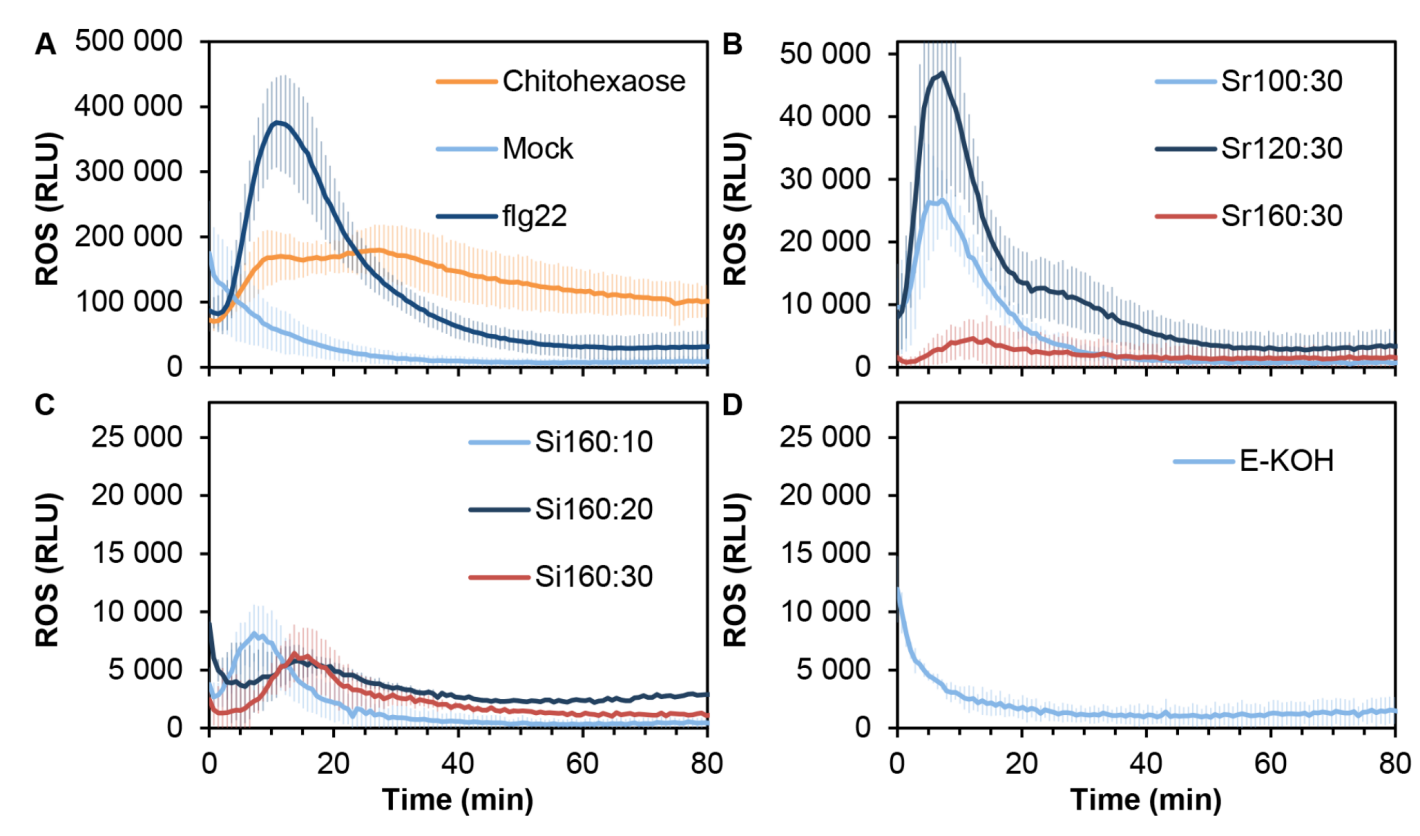
Reactive oxygen species production in *Arabidopsis thaliana* after treatment with *Equisetum arvense* cell wall fractions. ROS production was measured as relative luminescence units (RLU) over the time. *A. thaliana* leaf-discs were treated with **A** distilled water (Mock), 100 nM flagellin (flg22) and 50 µM Chitohexaose, **B** 0.25 mg/ml of SWE fractions obtained after a ramp of temperatures during 30 min at 100 ºC (Sr100:30), 120 ºC (Sr120:30) and 160 ºC (Sr160:30), **C** 0.25 mg/ml of SWE fractions obtained after at isothermal 160 ºC during 10 min (Si160:10), 20 min (Si160:20) and 30 min (Si160:30) fractions and **D** 0.25 mg/ml of 4% KOH fractionation (E-KOH). Data represent mean ± SD (n = 8 biological replicates) in all panels. Data shown are from one representative experiment of the three carried out

**Fig. 6:**
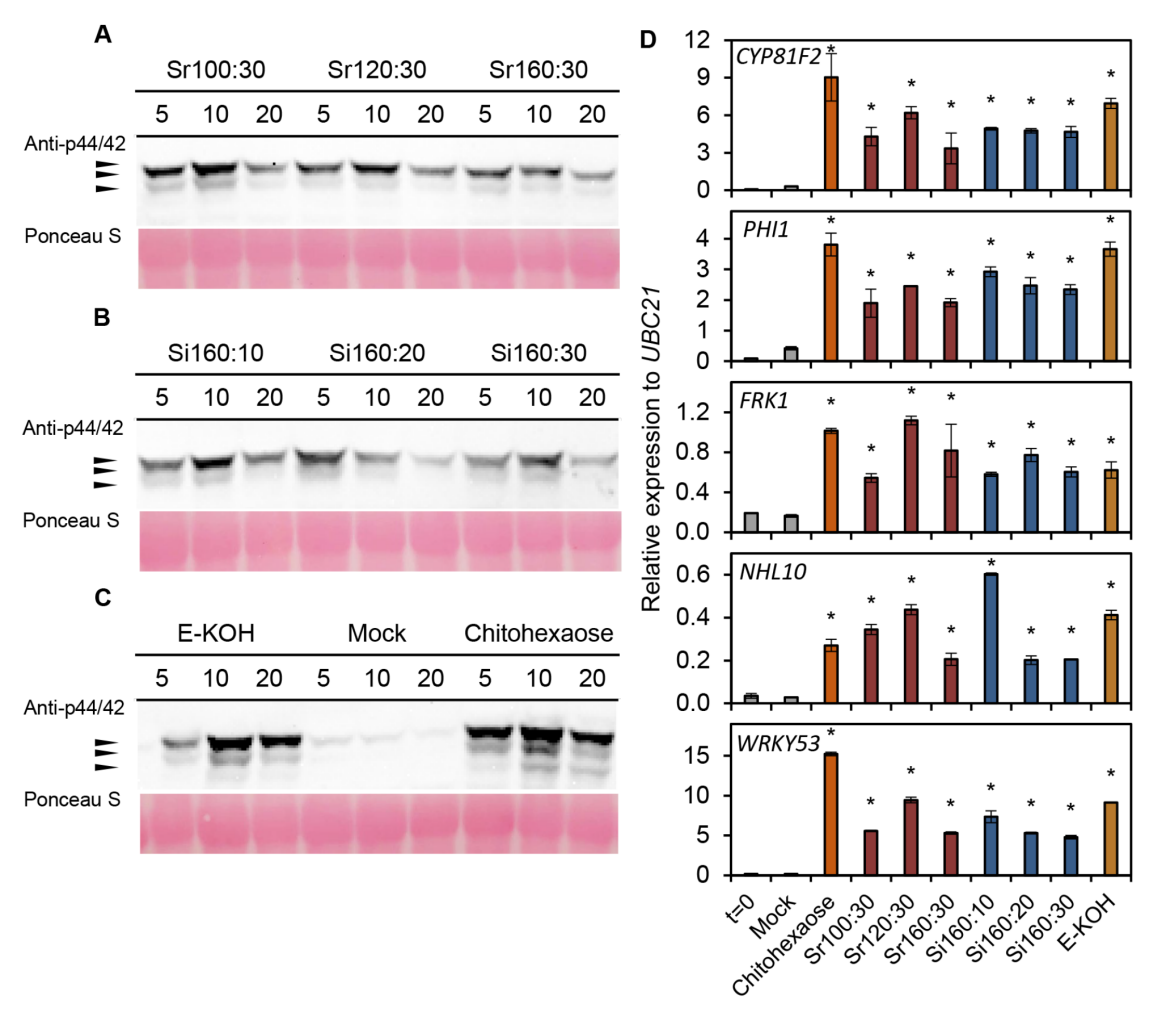
Pattern-triggered immunity hallmarks activation by *Equisetum arvense* cell wall fractions in *Arabidopsis thaliana* seedlings. MAPK phosphorylation was determined in 12-days old *A. thaliana* treated seedlings by Western Blot using anti-pTEpY antibody for phosphorylated MAPK moieties at different time points (5, 10 and 20 min) after the treatment with *E. arvense* cell wall fractions (0.25 mg/ml): **A** Sr fractions, **B** Si fractions and **C** E-KOH fraction, distilled water (mock) and 5 µM chitohexaose. Black arrows indicate the position of MPK6 (top), MPK3 (middle) and MPK4/11 (bottom) proteins. **D** Relative expression levels to *UBC21* (*At5g25769*) gene analysed by quantitative RT-PCR analysis in 12-days old *A. thaliana* seedlings at 30 min after each treatment. Data represent mean ± SE (n = 3 technical replicates). Statistically significant differences to mock according to Student’s t-test (* *p* < 0.01;). Data shown in all panels are from one experiment of the three performed that generated similar results

Next, we tested the capacity of SWE fractions of triggering other PTI hallmarks in *A. thaliana*, like ROS production, MAPK phosphorylation and immune-related gene expression up-regulation (Figs. 5 and 6). Regarding H_2_O_2_ production, SWE extracts triggered in the first 20 min after treatment a humbler ROS production than chitohexaose and flg22, used as positive controls, being the Sr fractions more active than Si (Fig. 5B, C). In particular Sr120:30 induced the most intense ROS burst. In contrast, E-KOH fraction, similarly to mock-treated plants, did not trigger H_2_O_2_ burst (Fig. 5D).

To further confirm the downstream immune activity of the SWEs, we next studied by Western blot the phosphorylation of mitogen-activated protein kinases (MPK3/MPK6/MPK4/MPK11) in *A. thaliana* seedlings treated with the different fractions and the controls (water and chitohexaose). MPK3 and MPK6 phosphorylation was detected at 5, 10 and 20 min after treatment with Sr and Si fractions (Fig. 6A-C). Of note, KOH fractions did also activate MPK3 and MPK6 phosphorylation at these time points despite such fractions did not trigger calcium influxes and ROS production (Figs. 3 and 5). In addition, MPK4/11-phosphorylation was only slightly detected in plants treated with KOH extraction and chitohexaose. Interestingly, while the MAPK phosphorylation peak was generally reached at 10 min after treatments, in plants treated with Si160:20 this peak appeared earlier (5 min; Fig. 6B). It should also be noted that SWE Sr100:30 and Si160:10 fractions, which showed the lowest signals in calcium influx assays, seemed to activate the highest MPK3 phosphorylation level, although the differences were very subtle and this assay should be considered only semi-quantitative. (Fig. 6A, B).

Activation of expression of PTI-marker genes (*CYP81F2*, *WRKY53, FRK1*, *PHI1*, *NHL10* and *PROPEP1*) was analysed by qRT-PCR in *A. thaliana* seedlings 30 min after treatment with SWE and E-KOH fractions. As shown in Fig. 6D, all the studied genes were significantly up-regulated in plants treated with all SWE and E-KOH fractions in comparison with mock-treated plants, and the level of gene up-regulation was similar to that observed in plants treated with chitohexaose (Fig. 6D), further confirming that all the extracted fractions contain DAMPs triggering PTI.

### SWE fractions enhance resistance in pepper plants against the pathogen *Sclerotinia sclerotiorum*

**Fig. 7:**
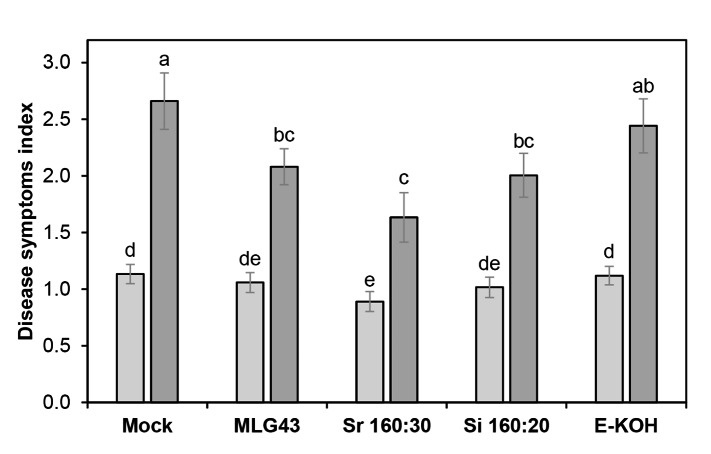
Treatment of pepper plants with subcritical water extractions of *Equisetum arvense* cell wall enhances disease resistance to *Sclerotinia sclerotiorum*. Plants were foliar-treated with 0.125 mg/ml of SWE fractions (Sr160:30 or Si160:20) or lichenase-digested E-KOH fraction 2 days prior pathogen inoculation. MLG43 at 0.125 mg/ml was used as positive control and distilled water as negative control (mock). Disease symptoms index produced by *Sclerotinia sclerotiorum* were quantified at 5 (light colour) and 9 dpi (dark colour) in leaves of pepper plants. Data represent mean ± SE (n = 24 biological replicates). Different letters indicate significant differences by Student’s t-test (*p* < 0.05). Disease experiments were performed four times and one representative experiment is shown

In order to check the potential of SWE fractions to enhance crops resistance to pathogens, one fraction from each SWE extraction sequence was selected to treat pepper plants 48 h before inoculating them with the fungal pathogen *S. sclerotiorum*. Sr160:30 and Si160:20 were chosen for these disease resistance analyses since they trigger the highest calcium bursts (Fig. 3B, C). Since E-KOH did not trigger calcium influxes (Fig. 3D), and we previously demonstrated that this fraction gained some DAMP activity when digested with lichenase (Rebaque et al. 2021), an enzyme that releases oligosaccharides from MLGs (Henrissat and Bairoch 1993), we decided to use E-KOH fraction digested with this enzyme for comparison in the protection assay, and we also include in the experiment for comparison the PTI-active ligand MLG43, that has been show to confer enhanced disease resistance against different pathogens in MLG43-treated *A. thaliana*, tomato and pepper (Rebaque et al. 2021). Interestingly, pepper plants pre-treatments with selected SWE fractions showed enhanced resistance against the fungal pathogen as revealed by a significant decrease of disease symptoms at 9 dpi in comparison to mock-treated plants (Fig. 7). In particular, disease symptom index decreased from 2.7 in mock-treated plants to 1.6 and 2 in Sr160:30- and Si160:20-treated plants. The reduction of disease symptoms in Sr160:30- and Si160:20-treated plants was quite similar than that observed in MLG43-treated plants (Fig. 7). In contrast, lichenase-digested E-KOH fraction was not able to significantly reduce the disease symptom index produced by *S. sclerotiorum* in pepper in comparison to mock-treated plants (Fig. 7).

## Discussion

The complexity and diversity of plant cell walls makes them a potential source of a wide range of molecules of different nature and with promising opportunities for multiple industrial applications (Carpita and McCann 2020; Engelberth et al. 2021; Rebaque et al. 2017; Rudjito et al. 2019; Yilmaz-Turan et al. 2020). For the purpose of obtaining plant cell wall-derived molecules (DAMPs) to develop novel crop protection products triggering plant immunity, classical chemical-based extractions present impediments that make them unfeasible. For instance, the resulting fractions must be neutralised, producing an enormous concentration of salts (> 50 g/l) that cannot be applied to the crop fields and that would have to be eliminated, generating large amounts of chemical wastes, and multiplying production costs.

In this work, we have explored SWE technology as an alternative to obtain cell wall fractions that can be directly used in the development of products to be used in agriculture. SWE has been already explored as a possible alternative technique to classical extractions to obtain cell wall polymers for different applications and has been successfully scaled-up from the laboratory to pilot industrial plants (Kilpeläinen et al. 2014; Rudjito et al. 2019; Yilmaz-Turan et al. 2020). Alkali extraction of cell wall fractions has been previously shown to give higher extraction yields than SWE although the latest present qualitative advantages (Yilmaz-Turan et al. 2020). Interestingly, our results show that similar extraction yields from *E. arvense* cell walls by SWE and alkali extraction can be obtained, but in the case of SWE using only water as solvent, avoiding neutralization steps, and shorting extraction times in comparison to those of alkali-based extractions. In this respect, according to our results, the most appropriate strategy seems to be to work at maximum temperature (160 ºC), since the last step of the Sr sequence (performed at 160 ºC) and the three Si fractions (extracted at 160 ºC), yielded highest proportions of sugars (Fig. 2B) and low proportion of proteins (Supplementary Fig. 1). The calcium burst triggered by the fractions obtained at shortest times or lowest temperatures seem to be weaker than those observed with SWE fractions obtained at longer times or higher temperatures (Figs. 3 and 4), which would indicate that to make a first cleaning extraction step could release compounds with low activity or that might inhibit calcium fluxes and ROS production. Although intracellular calcium fluxes and H_2_O_2_ bursts are two of the most commonly used hallmarks of plant immunity (Martín-Dacal et al. 2023; Ranf et al. 2015), in this study we also tested additional hallmarks like MAPK phosphorylation and marker gene up-regulation. Of note, E-KOH fraction that did not trigger ROS and calcium fluxes, and some Si fractions obtained at shortest times (Si160:10) or lowest temperatures (Sr100:30 and Sr120:30) that trigger low ROS production and calcium fluxes were found to activate PTI downstream processes, like MAPK phosphorylation and marker gene up-regulation (Figs. 5 and 6). This effect of E-KOH and these SWE fraction on PTI hallmarks deserves future work, since it is generally accepted that for these downstream PTI hallmarks to be activated (MAPK phosphorylation and gene expression), there must be an activation of at least one of the two upstream hallmarks (calcium and/or ROS) (Yu et al. 2017). These results also remark the relevance of testing different PTI hallmarks to identify active wall fractions triggering plant immunity.

The composition of the fractions obtained by SWE using water clearly differs from that obtained with alkali (Fig. 3). Previous works indicated that the cell wall of *E. arvense* is dominated by the presence of glucans (more than 50%), mainly cellulose and MLGs, with very little presence of xyloglucan, while the second most abundant component (almost 40%) is homogalacturonan (Fry et al. 2008; Sørensen et al. 2008; Xue and Fry 2012). Arabinose and galactose are also important components, which do not seem to form typical type II arabinogalactans, but galactans and arabinans in the form of pectin side chains, and glucuronoxylan-type hemicelluloses can also be detected, but in low proportions (Xue and Fry 2012). Our data indicate that SWE clearly extracts more pectic components (homogalacturonan, arabinans and galactans) than alkali extraction, which extracts mainly hemicelluloses (glucans, mannans and xylans; Fig. 3) as previously described for *E. arvense* and other sources of biomass (Le Normand et al. 2014). Although glucuronoarabinoxylans have been reported to be present in the wall of *E. arvense* (Xue and Fry 2012), the high proportions of glucuronic acid compared to xylose (Fig. 3) determined in SWE fractions pointed to the possible presence of long glucuronic acid chains rather than simple decorations of this monosaccharide in glucuronoarabinoxylans. Our data reveal that SWE of *E. arvense* walls targets mainly pectic polysaccharide populations, shifting from linear galacturonan-rich populations to more complex arabinan/galactan populations with increasing temperatures and times. In contrast, alkaline treatments target mainly hemicellulosic populations (probably mannans, xylans and β-glucans). This has strong significance to the valorisation of *E. arvense* biomass for different material applications, as the extraction process can be directed towards target pectin or hemicellulose populations depending on the intended application.

*E. arvense* KOH cell wall fraction has been previously described to be inactive triggering calcium influxes (Rebaque et al. 2021). Here we have corroborated that result, and we have also observed that it is not able to induce a ROS burst. Only when KOH fraction is lichenase-digested, the released MLG oligosaccharides trigger PTI responses (calcium influxes and ROS) in plants (Rebaque et al. 2021). In contrast, we have observed that “crude” SWE fractions (non-digested with enzymes) triggered the earliest PTI responses, such as calcium influxes and ROS bursts. Therefore, the SWE is yielding active molecules that were not obtained by classical alkali extractions. This differential activity of *E. arvense* KOH and SWE fractions could be explained in several ways, which are not mutually exclusive: (I) the simplest one is that molecules extracted with alkali could be less immunoactive per se than those extracted with water under subcritical conditions, which, as above indicated, have a pectic profile, which could be related to the presence of the well characterized DAMPs oligogalacturonides derived from pectins (Brutus et al. 2010; Côté and Hahn 1994; Davidson et al. 2017); (II) another simple (and probable) explanation, would be that despite using dialysis membranes with a molecular weight cutoff of 1 kDa (size of an hexose hexasaccharide), the putative immunoactive glycans (oligosaccharides of DP equal or lower than 6) could be being lost during this dialysis process, since partial degradation of carbohydrates upon SWE extraction into molecules of lower degree of polymerization has been already reported (Ruthes et al. 2017); and (III) the oxidation of reducing end of glycans by KOH could result in the modification of glycans three-dimensional structure and alteration of their perception as DAMPs by plant PRRs (Knill and Kennedy 2003). Indeed, the reduction of the activity of oxidized OGs and cellulose-derived oligosaccharides and chemically synthesised β-glucan structures containing an aminoalkyl motif at their reducing end has been previously described (Benedetti et al. 2018; Locci et al. 2019; Rebaque et al. 2021).

Finally, guided by the calcium influx data, we selected two of the best SWE fractions and compared them with the lichenase-digested E-KOH fraction. Our pepper disease resistance data with SWE fractions in comparison with lichenase-digested E-KOH fraction (Fig. 7), clearly indicated that SWE technology has the potential to be used for revalorising low-value plant biomass into added-value materials containing DAMPs. Both SWE fractions tested, unlike E-KOH, were able to significantly improve the natural protection of pepper plants against the fungal pathogen *S. sclerotiorum* (Fig. 7) similarly to the effect produced by the DAMP MLG43 (Rebaque et al. 2021). In conclusion, SWE technology has been proved here as a potential sustainable technology to obtain active molecules (DAMPs) from plant cell walls that can be used to develop products that enhance the resistance of plants against pathogens.

### Electronic supplementary material

Below is the link to the electronic supplementary material.

Supplementary Material 1

Supplementary Material 2

## Data Availability

Enquiries about data availability should be directed to the authors.
