# Supplementary material for: Subcritical water extraction of Equisetum arvense biomass withdraws cell wall fractions that trigger plant immune responses and disease resistance
Source: Plant Mol Biol. 2023 May 2;113(6):401–14. doi: 10.1007/s11103-023-01345-5 (PMC10730674; doi:10.1007/s11103-023-01345-5)
Supplement: Supplementary file 1 — Supplementary Material 1 [file 11103_2023_1345_MOESM1_ESM.pdf]

Figure S1

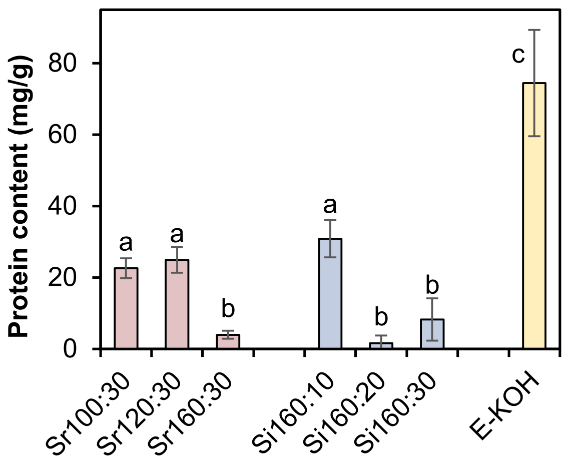

Fig. S1 ***Equisetum arvense*** **cell wall fractions protein quantification.** Total protein content, expressed as mg/g of total fraction, was determined in SWE fractions obtained after a ramp of temperatures [30 minutes at 100 ºC (Sr100:30), 30 minutes at 120 ºC (Sr120:30) and 30 minutes at 160 ºC (Sr160:30)], upon isothermal extraction [160 ºC 10 minutes (Si160:10), 20 minutes (Si160:20) and 30 minutes (Si160:30)], or chemical extraction (incubation with 4% KOH, E-KOH). Data represent mean ± SD. Different letters indicate significant differences by Student's t-test ($p < 0.05$).
