# Supplementary material for: Subcritical water extraction of Equisetum arvense biomass withdraws cell wall fractions that trigger plant immune responses and disease resistance
Source: Plant Mol Biol. 2023 May 2;113(6):401–14. doi: 10.1007/s11103-023-01345-5 (PMC10730674; doi:10.1007/s11103-023-01345-5)
Supplement: Supplementary file 2 — Supplementary Material 2 [file 11103_2023_1345_MOESM2_ESM.docx]

| **Table S1. Oligonucleotides used for qRT-PCR** | | | |
| --- | --- | --- | --- |
| ***Arabidopsis thaliana*** | | | |
| **Gene** | **Locus** | **Forward oligonucleotide** | **Reverse oligonucleotide** |
| *UBQ21* | *AT5G25760* | GCTCTTATCAAAGGACCTTCGG | CGAACTTGAGGAGGTTGCAAAG |
| *CYP81F2* | *AT5G57220* | TATTGTCCGCATGGTCACAGG | CCACTGTTGTCATTGATGTCCG |
| *WRKY53* | *AT4G23810* | CACCAGAGTCAAACCAGCCATTA | CTTTACCATCATCAAGCCCATCGG |
| *FRK1* | *AT2G19190* | ATCTTCGCTTGGAGCTTCTC | TGCAGCGCAAGGACTAGAG |
| *PHI1* | *AT1G35140* | TTGGTTTAGACGGGATGGTG | ACTCCAGTACAAGCCGATCC |
| *NHL10* | *AT2G35980* | TTCCTGTCCGTAACCCAAAC | CCCTCGTAGTAGGCATGAGC |
